# Developing a modular architecture for creation of rule-based clinical diagnostic criteria

**DOI:** 10.1186/s13040-016-0113-5

**Published:** 2016-10-21

**Authors:** Na Hong, Jyotishman Pathak, Christopher G. Chute, Guoqian Jiang

**Affiliations:** 1 Department of Health Sciences Research, Mayo Clinic, 200 First Street, SW, Rochester, MN 55905 USA; 2 Institute of Medical Information, Chinese Academy of Medical Sciences, Beijing, China; 3 Weill Cornell Medical College, Cornell University, New York, NY USA; 4 School of Medicine, Johns Hopkins University, Baltimore, MD USA

**Keywords:** Diagnostic Criteria, Ontology, ICD-11, QDM, SWRL

## Abstract

**Background:**

With recent advances in computerized patient records system, there is an urgent need for producing computable and standards-based clinical diagnostic criteria. Notably, constructing rule-based clinical diagnosis criteria has become one of the goals in the International Classification of Diseases (ICD)-11 revision. However, few studies have been done in building a unified architecture to support the need for diagnostic criteria computerization. In this study, we present a modular architecture for enabling the creation of rule-based clinical diagnostic criteria leveraging Semantic Web technologies.

**Methods and results:**

The architecture consists of two modules: an authoring module that utilizes a standards-based information model and a translation module that leverages Semantic Web Rule Language (SWRL). In a prototype implementation, we created a diagnostic criteria upper ontology (DCUO) that integrates ICD-11 content model with the Quality Data Model (QDM). Using the DCUO, we developed a transformation tool that converts QDM-based diagnostic criteria into Semantic Web Rule Language (SWRL) representation. We evaluated the domain coverage of the upper ontology model using randomly selected diagnostic criteria from broad domains (n = 20). We also tested the transformation algorithms using 6 QDM templates for ontology population and 15 QDM-based criteria data for rule generation. As the results, the first draft of DCUO contains 14 root classes, 21 subclasses, 6 object properties and 1 data property. Investigation Findings, and Signs and Symptoms are the two most commonly used element types. All 6 HQMF templates are successfully parsed and populated into their corresponding domain specific ontologies and 14 rules (93.3 %) passed the rule validation.

**Conclusion:**

Our efforts in developing and prototyping a modular architecture provide useful insight into how to build a scalable solution to support diagnostic criteria representation and computerization.

**Electronic supplementary material:**

The online version of this article (doi:10.1186/s13040-016-0113-5) contains supplementary material, which is available to authorized users.

## Background

### Introduction

Diagnostic criteria are one of the most valuable knowledge sources for supporting clinical decision-making and improving patient care [1–4]. The clinical informatics research community has been seeking a solution to standardize and computerize clinical diagnosis criteria for all clinical domains. Diagnostic criteria are usually scattered over different media such as medical textbooks, literatures and clinical practice guidelines mostly in textual format. Furthermore, they are usually described in different narrative style, granularity, term usage and inner logic. There is an urgent need to develop a standard information model specification and a unified architecture to support the diagnostic criteria modeling and representation, and thereby enabling computerization and machine interpretability. To achieve a unified architecture, the following aspects should be considered: a) an information model that supports standard representation of diagnostic criteria; b) semantic interoperability and expressivity of a knowledge representation language; c) rule-based reasoning capability over factual knowledge; and d) a standard exchange format for different layers of the architecture.

The content model of the International Classification of Diseases (ICD)-11 developed by the World Health Organization (WHO) is a structured framework that defines “a classification unit” in ICD in a standard way in terms of its components that allows computerization [5]. Under the definition of the content model, each ICD entity can be seen from different dimensions and there are currently 13 main dimensions in the content model. One purpose of the ICD-11 content model is to use different settings of these dimensions or parameters to construct different sets of diagnostic criteria, so different elements in the content model come together to define the diagnosis criteria of a particular ICD category. The ICD-11 content model entails a diagnostic criteria computerization at a high level and it has achieved consensus among the members in the ICD Revision Group, thus we regard it as a feasible framework for constructing a unified architecture for computable diagnostic criteria creation.

The Quality Data Model (QDM) [6] is an information model that describes clinical concepts in a standardized format to enable electronic quality-performance measurement in support of the Meaningful Use Program of the Health Information Technology for Economic and Clinical Health Act [7, 8]. It allows electronic clinical quality measure (eCQM) developers and many clinical researchers or performers to describe clearly and unambiguously the data required to calculate the performance measure. QDM enables electronic health records (EHR) and other clinical electronic system to share a common understanding and interpretation of the clinical data. To describe different parts of the clinical care process, QDM defines many datatypes to specify the context in which each category is used. As a QDM serialization format, the Health Quality Measure Format (HQMF) [9] is a HL7 standard that formally represents a eCQM (data elements, logic, definitions, etc.) as an electronic document to support consistent and unambiguous interpretation.

Semantic Web technologies provide a homogeneous framework that enables an ontology-based modeling with the Web Ontology Language (OWL) [10] and supports rule-based reasoning with the Semantic Web Rule Language (SWRL) [11]. In a semantic web environment, OWL is a W3C recommendation for ontology description and modeling and SWRL is a rule language to formalize and represent rules to support knowledge representation and reasoning. In the present study, we utilize and evaluate OWL and SWRL-based representation languages for formalizing complex inner logic for diagnostic criteria.

The objective of our study is to develop and evaluate a modular architecture for enabling the creation of rule-based clinical diagnostic criteria leveraging Semantic Web technologies. We prototype and evaluate a number of key components of the architecture, including an upper ontology and a transformation tool. We select a collection of QDM datatypes that are commonly used in describing diagnostic criteria and integrated them into ICD-11 Content Model for building an upper ontology. We perform the rule-based criteria translation and interaction following the HQMF standard format and propose extensions where needed.

### Related work

Previous studies have been conducted in integrating and formally expressing diagnostic rules from different perspectives. These rules are usually extracted from free-text-based clinical guidelines or diagnostic criteria, and integrated into computerized decision support systems to improve clinical performance and patient outcomes [12, 13]. The related studies mainly include as follows.

1. Clinical guideline computerization and Computer Interpretable Guideline (CIG) Systems. Various computerized clinical guidelines and decision support systems that incorporate clinical guidelines have been developed. Researchers have tried different approaches on computerization of clinical practice guidelines [12, 14–18]. Since guidelines cover many complex medical procedures, the application of computerized guideline in real-world practice is still very limited. However, the methods used to computerize guidelines are valuable in tackling the issues in diagnostic criteria computerization.
2. Formalization method studies on clinical research data. Previous studies investigated the eligibility criteria in clinical trial protocol and developed approaches for eligibility criteria extraction and semantic representation, and used hierarchical clustering for dynamic categorization of such criteria [19]. For example, EliXR provided a corpus-based knowledge acquisition framework that used the Unified Medical Language System (UMLS) to standardize eligibility-concept encoding and to enrich eligibility-concept relations for clinical research eligibility criteria from text [20]. QDM-based phenotyping methods used for identification of patient cohorts from EHR data also provide valuable reference for our work [21].

However, few studies are directly related to building a unified architecture to support the goal of diagnostic criteria formalization. In particular, the lack of a standards-based information model has been recognized as a major barrier for achieving computable diagnostic criteria [22]. Fortunately, current efforts in the development of international recommendation standard models in clinical domains provide valuable references for modeling and representing computable diagnostic criteria. The notable examples include the ICD-11 content model [5, 23] and the National Quality Forum (NQF) QDM [21, 24, 25].

## Methods

### Materials

#### WHO ICD-11 content model

WHO developed a content model to represent the knowledge that underlies the definition of an ICD entity [23]. The content model is composed of three layers: a foundation layer, a linearization layer, and an ontological layer. The foundation layer is the core product of the ICD-11 revision that stores the full range of knowledge of all classification units in ICD. Each ICD entity can be seen from different dimensions. The content model represents each one of these dimensions as a parameter. Currently, there are 13 main parameters defined in the content model to describe a category in ICD-11, as shown in Table 1 [5]. “Diagnostic Criteria” is one of the main parameters for describing an ICD category.

**Table 1:** The ICD11 Content Model Main Parameters

1. ICD Entity Title	
2. Classification Properties	
3. Textual Definitions	
4. Terms	
5. Body System/Structure Description	
6. Temporal Properties	
7. Severity of Subtypes Properties	
8. Manifestation Properties	
9. Causal Properties	
10. Functioning Properties	
11. Specific Condition Properties	
12. Treatment Properties	
13. Diagnostic Criteria	

#### NQF QDM

QDM consists of two modules: a data-model module and a logic module. The data-model module includes the notions of category (e.g., Medication), datatype (e.g., “Medication, Administered”), attribute (e.g., information about dosage, route, strength, and duration of a medication), and value set comprising concept codes from one or more terminologies. The logic module includes logic operators, functions, comparison operators, temporal operators, subset operators. As mentioned above, the HQMF provides a standard format to render the QDM-based criteria (i.e., instance data) in XML format using a collection of templates. In a previous study [26], we evaluated the feasibility of using QDM for representing diagnostic criteria through a data-driven approach and suggested that common patterns informed by QDM are useful and feasible in building a standards-based information model for computable diagnostic criteria. In this study, we used the common patterns and selected a collection of QDM datatypes and attributes for developing an upper ontology.

### System architecture

**Fig. 1:**
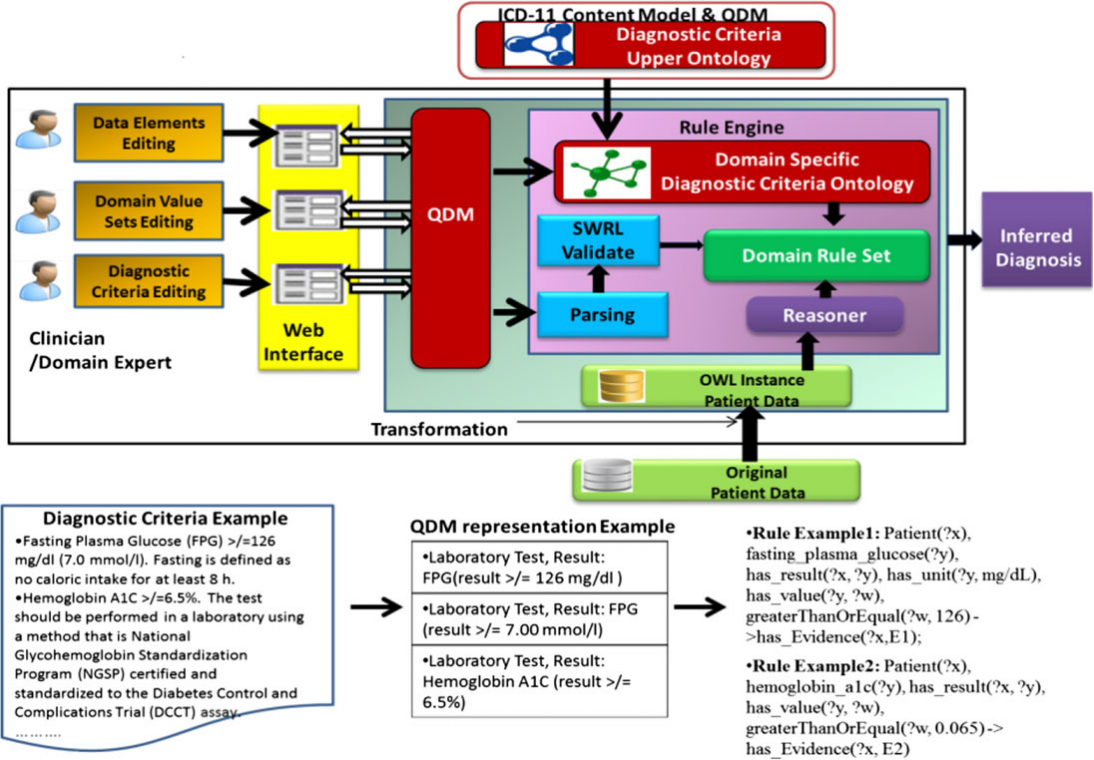
Overall System Architecture for Creation of Rule-based Clinical Diagnosis Criteria

The overall system architecture for the creation of rule-based clinical diagnosis criteria is shown in Fig. 1. The system architecture contains two major modules: one is an authoring module that utilizes a standards-based information model and the other is a translation module that leverages SWRL. The first module of the architecture contains an upper ontology that supports the element organization of diagnostic criteria. We integrated a collection of manually selected ICD-11 content model elements and QDM elements informed by the analysis of real-world diagnostic criteria [26]. The first module also contains a unified web user interface that supports collecting and authoring diagnostic criteria by clinicians or subject matter experts. Standard QDM model serves as a foundation layer for the downstream translation and reasoning. All collected data elements, value sets and logic expressions of diagnostic criteria are formalized using QDM-based HQMF templates. The second module of the architecture contains a rule translation engine that converts diagnostic criteria represented in QDM/HQMF format into a domain-specific diagnostic criteria ontology and a set of rules using SWRL.

### Developing a standard-based diagnostic criteria upper ontology

The purpose here is to integrate existing standard information models relevant to modeling of diagnostic criteria by expert review and manual editing. As previously mentioned, we choose the ICD-11 content model and NQF QDM as reference information models. Our work in this stage is to create the diagnostic criteria upper ontology (DCUO) through the integration of ICD-11 content model with those QDM elements commonly used in diagnostic criteria. We evaluated the distribution of the QDM elements using a collection of textual diagnostic criteria. The selection of these QDM elements was informed by the results from a previous study [26]. We selected 10 QDM datatypes and 4 QDM attributes and integrated them with ICD-11 content model-based ontology schema. Table 2 shows a list of the QDM datatypes and attributes used for the integration. We used Protégé ontology editing environment [27] for manually integrating these two standard information models into a diagnostic criteria upper ontology.

**Table 2:** A list of selected QDM datatypes and attributes for developing the upper ontology

QDM Datatypes	QDM Attributes
Laboratory Test, Result	Result
Diagnostic Study, Performed	Method
Diagnostic, Active	Reason
Physical Exam, Performed	Severity
Symptom, Active	
Medication, Active	
Patient Characteristic Birth Date	
Patient Characteristic Race	
Patient Characteristic Sex	
Procedure, Recommended	

We merged these two information models manually by conducting both concept and property analysis. Specifically, ICD-11 content model contributes a well-defined concept schema with general concepts that provide a systematic view of disease, whereas the QDM contributes more specific elements of disease diagnosis. Therefore, we chose all classes defined in the ICD-11 content model as our fundamental classes of DCUO, and named them by a prefix “ICD:” in conjunction with its original class name. QDM datatypes (with a prefix “QDM”) as the subclasses merged with ICD-11 content model classes. The mappings of the merged classes and integrated properties are shown in the Table 3. In total, four QDM attributes are integrated into the DCUO as object properties [26]. In addition, we also defined a number of new classes in the DCUO so that they can be used for supporting the SWRL rule construction and reasoning. Currently DCUO includes 3 newly defined classes (with a prefix “DCUO”): Patient, Unit, and Evidence.

**Table 3:** The Concept Integration between QDM Datatypes and ICD-11 Content Model Elements

QDM Datatype Predicate	ICD-11 Content Model Element
Laboratory Test, Result owl:subClassOf	Investigation Findings
Diagnostic Study, Performed owl:subClassOf	Investigation Findings
Diagnostic, Active owl:subClassOf	Title
Physical Exam, Performed owl:subClassOf	Investigation Findings
Symptom, Active owl:subClassOf	Signs and Symptoms
Medication, Active owl:subClassOf	Treatment Properties
Patient Characteristic Birth Date owl:subClassOf	Specific Condition Properties
Patient Characteristic Race owl:subClassOf	Specific Condition Properties
Patient Characteristic Sex owl:subClassOf	Specific Condition Properties
Procedure, Recommended owl:subClassOf	Treatment Properties

### Transforming QDM templates into domain-specific diagnostic criteria ontology

The DCUO provides a schema of general diagnostic criteria representation. To build a scalable diagnostic rule translation environment, it is important to dynamically populate the DCUO with domain-specific elements and produce a Diagnostic Criteria Domain Ontology (DCDO) for a specific disease or condition, e.g. the DCDO for AMI (Acute Myocardial Infarction). DCDO extends the DCUO by adding the disease-specific sub-classes, attributes and instances, and the number of elements incorporated in a DCDO depends on the complexity of the diagnosis criteria. For example, the QDM elements extracted from the AMI diagnostic criteria are shown in an Supplementary File [see Additional file 1]. Each QDM element can be represented in a structured format using one of the HQMF templates [see Additional file 2]

To transform each QDM/HQMF template into a DCDO, we designed rule-based pipelines that support data extraction from diagnostic criteria encapsulated by a template. The parsing tool decomposed a HQMF template into different parts and populated the parsed elements into the DCDO that supports for the representation of a particular ICD disease. Therefore, to create a domain-specific DCDO, it requires three kinds of input data: the DCUO, a group of disease diagnosis criteria rendered in the QDM/HQMF templates, and a group of parsing rules for these templates. As previously mentioned, the DCUO is an upper-ontology that we created for general use and is applicable for all diagnostic criteria. And the rule-based HQMF XML template parsing and populating are core tasks in this transforming implementation. For example, Table 4 shows the QDM/HQMF templates we used to develop an AMI DCDO.

**Table 4:** HQMF templates used to represent the AMI diagnostic criteria

Template Name	Template ID	Template Structure	AMI diagnosis evidence
Laboratory Test, Result	2.16.840.1.1138 83.3.560.1.12	See Supplementary File 2, (1)	Biochemical markers Test, Cardiac biomarker cTn Test
Symptom, Active	2.16.840.1.1138 83.3.560.1.69	See Additional file 2, (2)	Ischemic symptoms Myocaridal ischemia
Diagnostic Study, Performed	2.16.840.1.1138 83.3.560.1.3	See Additional file 2, (3)	ECG Image Study Angiography Autopsy

According to the structured representation of the QDM/HQMF templates, the general rules to populate DCDO from the HQMF templates are:

1. Locate the class of {$datatypeName}, such as “Laboratory Test, Result”;
2. Extract $valueSetName from HQMF XML tag < title > “{$datatypeName}: {$valueSetName}” </title>;
3. Insert $valueSetName into DCUO as the subclass of this class, for example, “cTn Test” as the subclass of “Laboratory Test, Result”;
4. Extract properties elements from HQMF structured templates;
5. Insert properties into DCUO as the annotation properties of this class.

**Fig. 2:**
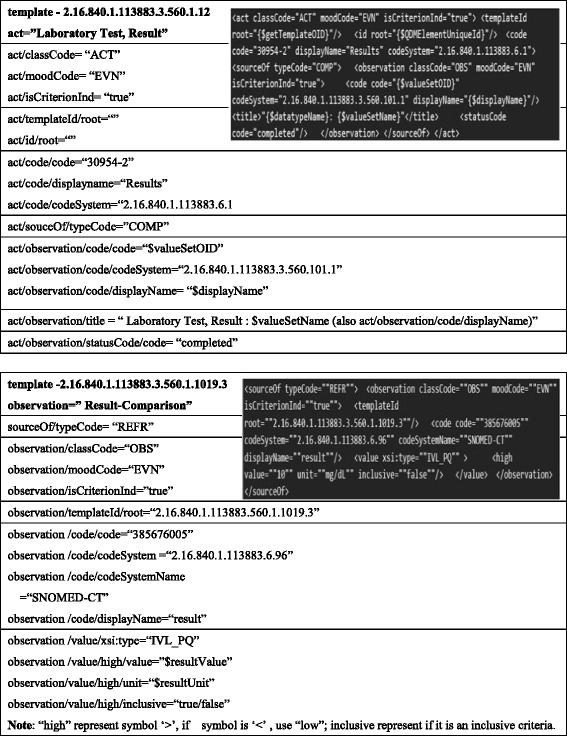
XML Parsing of the HQMF template “Laboratory Test, Result” (hqmf r1 template - 2.16.840.1.113883.3.560.1.12) and “Result-Comparison” (hqmf r1 template - 2.16.840.1.113883.3.560.1.1019.3)

Specifically, we analyzed the structure of HQMF templates, and developed a parsing tool to extract elements of each template. Figure 2 shows two HQMF template examples and their parsing rules. The upper part of Fig. 2 is the parsing of the Laboratory template “Laboratory Test, Result” (hqmf r1 template - 2.16.840.1.113883.3.560.1.12) and the lower part is the parsing of the Transfer of Care template “result-comparison” (hqmf r1 template - 2.16.840.1.113883.3.560.1.1019.3). Other templates (not shown) are parsed by a similar approach.

And then, we created mapping rules between the extracted XML elements and the elements in the DCUO ontology. Table 5 shows the mappings rules of two HQMF templates (2.16.840.1.113883.3.560.1.12 and 2.16.840.1.113883.3.560.1.1019.3). After populating the extracted elements into the DCUO, a domain-specific DCDO is produced [see Additional file 3]. The dynamics of DCDO are reflected by the variables of a template, such as “$valueSetOID”, and “displayName”. There are also a number of constants that maintain the metadata of each template.

**Table 5:** Integrating elements of HQMF template “Laboratory Test, Result” into DCUO

Template ID	Elements of template	Merge to DCUO
2.16.840.1.113883.3.560.1.12	“30954-2”	Annotation property of “Laboratory Test, Result”
	“Results”	Annotation property of “Laboratory Test, Result”
	“2.16.840.1.113883.6.1”	Annotation property of “Laboratory Test, Result”
	“$valueSetOID”	Annotation property of “$valueSetName”
	“2.16.840.1.113883.3.560.101.1”	Annotation property of “$valueSetName”
	“$displayName”	Annotation property of “$valueSetName”
2.16.840.1.113883.3.560.1.1019.3	“result-comparison”	Object Property: SubProperty of “has_result”
	“385676005”	Annotation property of “has_result”
	“2.16.840.1.113883.6.96”	Annotation property of “has_result”
	“SNOMED-CT”	Annotation property of “has_result”
	“result”	Annotation property of “has_result”
	“IVL_PQ”	Annotation property of “has_result_comparison”
	“$resultValue”	Value of Data Property “has_value”
	“$resultUnit”	Individual: Individual of class “Unit”

### Automatic rule composition and validation

After having a DCDO ontology produced, we developed JAVA-based algorithms using Protégé OWL API and SWRL API for conducting automatic rule composition and rule validation. These APIs are also responsible for rule assembling and rule grammar checking.

The SWRL syntax contains two parts: Body and Head. The Body part is also called the antecedent and the Head part is the consequent of the rule. There are 7 atom types that can be used as the components of the Body and Head: class, individual property, same individual, different individual, data valued property, built-in atom and data range [28].

Adhering to the SWRL structure and grammar, we designed a collection of translation algorithms that automatically extract the SWRL rule elements from the logic components of an HQMF XML template and then assemble these rule elements into the SWRL syntax.

For example, Table 6 shows the HQMF XML representation of a QDM-based criterion “Laboratory Test, Result: LDL-c (result < 100 mg/dL)”. The criterion is composed by two HQMF templates: the template “Laboratory Test, Result” (hqmf r1 template - 2.16.840.1.113883.3.560.1.12) and the template “result comparison” (hqmf r1 comparison template - 2.16.840.1.113883.3.560.1.1019.3). Our translation algorithms then automatically extract the SWRL rule elements from the logic components of the two templates and then assemble these rule elements into the SWRL syntax. The translation processing is shown in Table 7.

**Table 6:** The example of HQMF XML representation of the QDM-based criterion

The HQMF XML representation of the QDM-based criterion “Laboratory Test, Result: LDL-c (result < 100 mg/dL)”
<!-- Laboratory Test, Result pattern --> <templateId root = "2.16.840.1.113883.3.560.1.12"/> <id root = "c5244e91-3c2e-4863-ae87-a48556b9e3ae"/> <code code = "30954-2" displayName = "Results" codeSystem = "2.16.840.1.113883.6.1"/> <sourceOf typeCode = "COMP"> <observation classCode = "OBS" moodCode = "EVN" isCriterionInd = "true"> <code code = "2.16.840.1.113883.3.117.1.7.1.215" displayName = "LDL-c LOINC Value Set" codeSystem = "2.16.840.1.113883.3.560.101.1"/> <title > Laboratory Test, Result: LDL-c (result &lt; 100 mg/dL)</title> <statusCode code = "completed"/> <sourceOf typeCode = "REFR"> <observation classCode = "OBS" moodCode = "EVN" isCriterionInd = "true" > <templateId root = "2.16.840.1.113883.3.560.1.1019.3"/> <code code = "385676005" codeSystem = "2.16.840.1.113883.6.96" displayName = "result" codeSystemName = "SNOMED-CT"/> <value xsi:type = "IVL_PQ"> <high value = "100" unit = "mg/dL" inclusive = "false"/> </value> </observation> </sourceOf> </observation> </sourceOf>

**Table 7:** The Steps for Translating Individual Criteria to a SWRL Rule

The Steps and the Composed SWRL Rule	
Step 1: Insert the element “LDL-c” into DCDO as a subclass of the class “Laboratory Test, Result”. Step 2: Insert the element “mg/dL” as an individual of the class “Unit”, and insert the element “ev1” as an individual of the class “Evidence”. Step 3: The elements extracted from the templates are represented into the following 4 types of the SWRL atom: (1) Class atom : Patient(?x), LDL-c(?y) (2) Individual property atom: has_result_comparison(?x, ?y), has_unit(?y, mg/dL) (3) Data valued property atom: has_value(?y, ?z) (4) Built-in atom: lessThan(?z, 100) (5) Data range atom: xsd:int(?z) Step 4: Compose the atoms into a SWRL rule: Rule:Patient(?x),LDL-c(?y),has_result_comparison(?x, ?y),has_value(?y, ?z), int(?z),has_unit(?y, mg/dL),lessThan(?z, 100)- > has_evidence(?x,ev1)	

Each rule is generated from an individual criterion. After each rule is composed, our algorithms could aggregate these individual rules with logic operators for the diagnosis reasoning.

### Evaluation design

First, we evaluated the domain coverage of the ICD-11 content model for representing diagnostic criteria. We collected 20 diagnostic criteria from different clinical topics and manually annotated them with the elements in the ICD-11 content model.

Second, we evaluated the translation algorithms in the following two aspects: ontology population and rule generation. Currently,we tested the ontology population algorithms using 6 HQMF templates. The 6 HQMF templates are listed in Table 8. The first author (NH) assessed whether they are correctly parsed and represented in the domain ontology, and the assessment results were verified by the other three co-authors.

**Table 8:** A list of the HQMF templates used for the evaluation

HQMF templates	
1) “Laboratory Test, Result” (hqmf r1 template - 2.16.840.1.113883.3.560.1.12) 2) “Patient Characteristic Sex”(hqmf r1 template - 2.16.840.1.113883.3.560.1.402) 3) “Patient Characteristic Birth Date”(hqmf r1 template - 2.16.840.1.113883.3.560.1.400) 4) “result/is present”(hqmf r1 template - 2.16.840.1.113883.3.560.1.1019.1) 5) “result/valueset”(hqmf r1 template - 2.16.840.1.113883.3.560.1.1019.2) 6) “result/comparison” (hqmf r1 template - 2.16.840.1.113883.3.560.1.1019.3)	

We then tested the rule generation algorithms using 15 QDM-based criteria represented in HQMF XML format. All the 15 criteria are selected from existing clinical quality measures [29] that use the HQMF template - “Laboratory Test, Result” (hqmf r1 template - 2.16.840.1.113883.3.560.1.12). We used Protégé SWRL API to validate the syntactical correctness of the generated SWRL rules. The first author assessed the semantic correctness of the generated SWRL rules through comparing the HQMF XML-based logic with SWRL rule logic and the assessment results were verified by the other three co-authors.

## Results

### DCUO development and evaluation

**Fig. 3:**
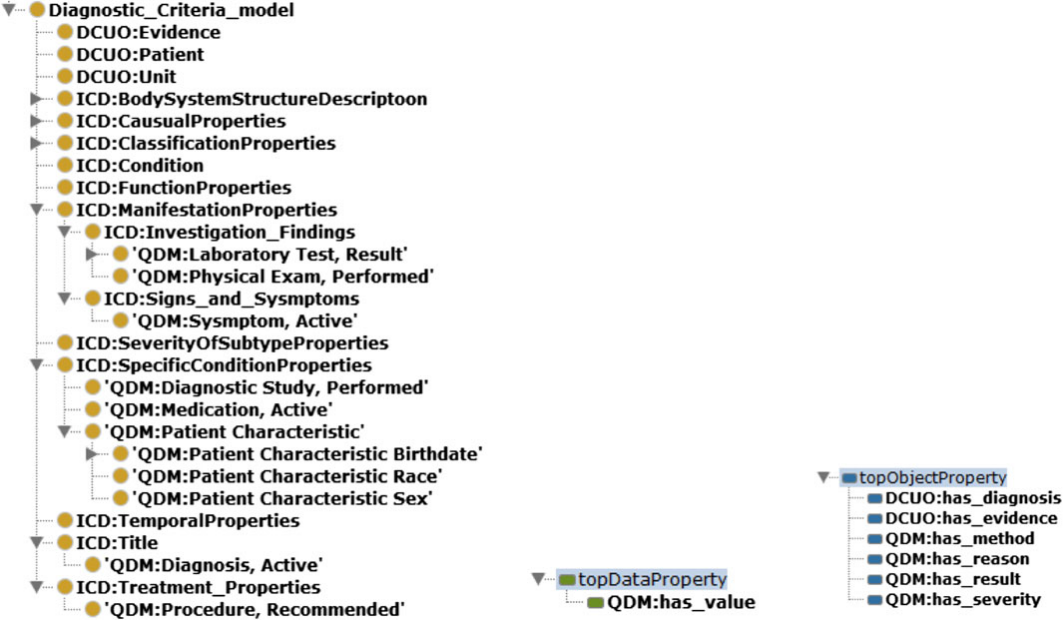
The Protégé Screenshots for the Diagnostic Criteria Upper Ontology (DCUO)

Figure 3 shows a screenshot of the classes and properties integrated in the DCUO in Protégé ontology editing environment. There are 14 root classes, 21 subclasses, 6 object properties and 1 data property in the ontology. In this ontology, 22 classes came from the ICD-11 content model with the namespace prefix ‘ICD’, 10 of the classes are integrated from the QDM datatypes with the namespace prefix ‘QDM’ and 3 classes with the namespace prefix ‘DCUO’ created for the need of representing diagnostic criteria in the SWRL rules.

We also evaluated the domain coverage of ICD-11 content model elements in annotating diagnostic criteria. Table 9 shows distribution of annotations based on ICD-11 content model elements. The results showed that Investigation Findings, and Signs and Symptoms are the two most commonly used element types in diagnostic criteria description. The results are consistent with the analysis we did for the QDM elements in a previous study [26].

**Table 9:** Distribution of annotations based on ICD-11 Content Model Elements

ICD-11 content model element	Count	Examples
Investigation Findings	74	Serum triglycerides
Sign and Symptom	69	Fatigue, Headache
Title	20	Metabolic Syndrome
Causal Properties	18	Pericardial effusion
Classification	12	T71
Severity Of Subtype	10	Mind, Moderate, Severe
Body System/Structure	8	Nervous system
Specific Condition	3	Female, Pregnancy
Temporary Properties	2	Age 55, sudden

### Translation algorithms evaluation

To extract elements in each HQMF template, we developed a collection of JAVA-based XML parsing and mapping algorithms. The algorithms automatically extract elements from HQMF templates and convert them into corresponding DCDO elements. As the evaluation results, all 6 HQMF templates are successfully parsed and populated into their corresponding DCDO ontologies. Human-based review confirmed that the elements in the templates are correctly represented in the target ontology.

For the rule generation algorithm evaluation, in total, 15 SWRL rules were generated from 15 QDM/HQMF-based individual criteria. Table 10 shows a list of the 15 QDM/HQMF-based criteria and the syntactic validation results using the Protégé SWRL validation tool. Of them, 14 rules (93.3 %) passed the rule validation whereas one rule (6.7 %) failed to pass. Human-based review analysis found that the failure was caused by an invalid expression ‘[copies]/mL’ that contains special characters ‘[’ and ‘]’. Human-based review also confirmed the semantic correctness of all 15 generated rules.

**Table 10:** A list of 15 QDM/HQMF-based criteria and the validation results

QDM/HQMF-based Criteria Using HQMF Template - “Laboratory Test, Result” (hqmf r1 template - 2.16.840.1.113883.3.560.1.12)	If passed rule syntax validation?
Laboratory Test, Result: INR (result > = 2)	Yes
Laboratory Test, Result: Hospital Measures-Neutrophil count (result < 500 per mm3)	Yes
Laboratory Test, Result: High Density Lipoprotein (HDL) (result < 40 mg/dL)	Yes
Laboratory Test, Result: Hepatitis A Antigen Test (result: 'Seropositive')	Yes
Laboratory Test, Result: Hepatitis B Antigen Test (result: 'Seropositive')	Yes
Laboratory Test, Result: HIV Viral Load (result < 200 copies/mL)	No
Occurrence A of Laboratory Test, Result: High Density Lipoprotein (HDL) (result < 60 mg/dL)	Yes
Occurrence A of Laboratory Test, Result: LDL Code (result < 100 mg/dL)	Yes
Occurrence A of Laboratory Test, Result: LDL-C Laboratory Test (result < 100 mg/dL)	Yes
Laboratory Test, Result: Macroalbumin Test (result: 'Positive Finding')	Yes
Laboratory Test, Result: Mumps Antigen Test (result: 'Seropositive')	Yes
Laboratory Test, Result: Prostate Specific Antigen Test (result < = 10 ng/mL)	Yes
Laboratory Test, Result: Measles Antigen Test (result: 'Seropositive')	Yes
Laboratory Test, Result: Rubella Antigen Test (result: 'Seropositive')	Yes
Laboratory Test, Result: High Density Lipoprotein (HDL) (result < 40 mg/dL)	Yes

### Implementation of a translation pipeline prototype

**Fig. 4:**
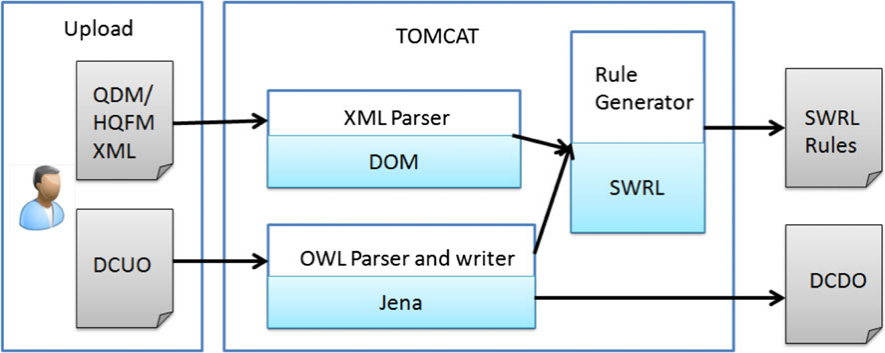
A Pipeline of SWRL Rule Generation

**Fig. 5:**
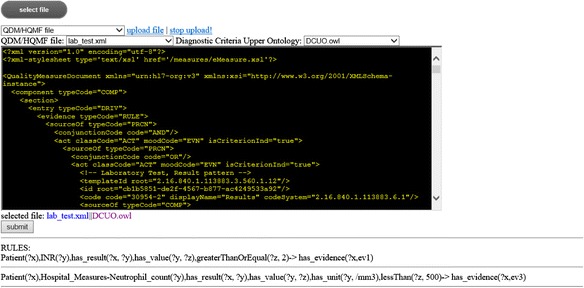
A Prototype Implementation of a HQMF to SWRL Translation Pipeline with a Web Interface

For implementing our rule generation algorithms, we assembled a computational pipeline (See Fig. 4). A prototype of a web-based interface is developed for the pipeline as shown in Fig. 5. The prototype allows users to upload a QDM/HQMF file and the DCUO file, and generate a collection of corresponding SWRL rules.

## Discussion

There are three main contributions in this study. First, we created two types of ontologies (DCUO and DCDO) to build a flexible knowledge representation framework for representing clinical diagnostic criteria. Second, we developed automatic rule translation methods that are built on standard data model and data structure. This will ensure that the system is extensible and can be used to enable extensive support for representation and computation of diversified diagnostic criteria. Third, system architecture supports reuse of existing standards from the perspectives of information model, terminology services and technical interface.

In this study, we developed a modular architecture to support the authoring and formalization of diagnostic criteria knowledge leveraging Semantic Web OWL and SWRL technologies. Semantic Web technologies provide a scalable framework for standards-based knowledge representation and reasoning and could support the diagnostic criteria representation in a machine-readable and interpretable way. The diagnostic criteria upper ontology and domain-specific ontology are all represented in OWL which provides a foundation for coding SWRL rules. And the rules generated from QDM HQMF-based criteria are formalized and represented in SWRL that leverages the full reasoning power of OWL DL when invoking a rule engine.

For the DCUO building module, we integrated the elements of the ICD-11 content model and the QDM model based on the investigation of the concept and attribute distribution of real-world diagnostic criteria. The elements used to build DCUO are evaluated by our analysis on a number of randomly selected diagnostic criteria collected from a variety of domains. Through a manual integration, we built a fundamental knowledge organization schema that can be used to support representing diagnostic criteria for a particular domain. Current editing work is performed in the Protégé editor environment. We plan to release the DCUO ontology through the National Center for Biomedical Ontology BioPortal [30] for the community-based review and feedback.

For the rule translation engine module, the QDM-based HQMF templates are used as an intermediate standard interface that supports the communication between DCUO and those elements (e.g., individuals, standard codes and logic rules) that exist in the HQMF XML diagnostic criteria. All the rule atoms are parsed and extracted from an HQMF XML file and composed into the SWRL rules, and a DCDO is populated from the instance data encapsulated in the HQMF XML file. These generated rules and DCUO could be aggregated and used for reasoning against patient data for a particular disease in the future. Currently, our rules are evaluated on a particular QDM datatype (i.e., Laboratory Test, Result) that involves 6 HQMF templates, and the results show that the accuracy of the translation is above 90 %. Using the same principle, we consider that the HQMF parsing algorithms could achieve similar performance on the QDM datatypes in other QDM categories such as Symptom, Patient Characteristic, Diagnostic Study.

From the perspective of data standardization, following the rationale of the ICD-11 content model, the full range of different values for a given parameter would be predefined using standard terminologies and ontologies. In this study, the QDM-based criteria extracted from the CQMs took advantage of the predefined value sets in the National Library of Medicine Value Set Authority Center (VSAC)^1^. During our translation work, the value set metadata are automatically parsed and extracted from the HQMF XML file. Considering current VSAC does not include all the value sets associated with diagnostic criteria, our system architecture will support the extension of value set definitions in the future. In addition, we also plan to extend the web-based application functionalities as follows: 1) Ontology display and update; 2) Diagnostic criteria authoring by clinicians and domain experts, including value set services invoking and semi-automatic workflow for criteria editing.

There are a number of limitations in this study since the present study is mainly focused the feasibility of our proposed architecture. First, the DCUO (Diagnostic Criteria Upper Ontology) was reviewed for consensus and quality assurance only by a relatively small group (i.e., four authors). In the future, a rigorous ontology evaluation by a panel of experts from relevant domains will be useful in achieving consensus in terms of the vocabulary, syntax, structure, semantics, representation and context of the DCUO. We plan to use ontology evaluation methods as described by Vrandečić [31]. Second, we have not considered all complex conditions and details in the modeling of diagnostic criteria. For instance, the following problems need to be further considered.

- In the QDM model, the semantics of some templates are not expressed explicitly. For example, the QDM element ‘Patient Characteristic Birth Date’ is used to represent the numeric value comparison of the variable “Patient Age” (e.g. <low value=’18’ unit=’a’ inclusive=’true’/>), assuming the value of the variable “Patient Age” could be derived from the ‘Patient Characteristic, Birth Date’.
- In the present study, we have implemented translation algorithms only on a limited number (n = 6) of HQMF templates and the preliminary evaluation demonstrated that the translation performed is reasonably well. However, in total, there are 186 HQMF templates from diverse domains and these HQMF templates are updated continuously, so maintaining the transportability and reusability of the translation algorithms will be a challenge.
- For the diagnostic criteria rule generation using SWRL, the inclusion criteria are well supported by the built-in rule grammars, such as: comparison, mathematical functions, booleans, string and date/time. We understand that some of exclusion criteria could not be explicitly expressed in SWRL due to that negated atoms or disjunctions are not supported in SWRL.

In the future, we plan to improve our work from the following aspects: 1) to improve the system functions such as the DCDO enrichment, rule generation and computerized criteria display and execution; 2) to enhance diagnostic criteria annotations using the natural language processing (NLP) so that large amount number of diagnostic criteria text can be transformed into QDM-based structured format in a semi-automatic approach; 3) to execute diagnostic criteria against large scale patient data for target diseases.

## Conclusions

In this study, we developed a modular architecture for the creation of rule-based diagnostic criteria. We demonstrated the feasibility of prototyping a number of key components of our proposed architecture for diagnostic criteria knowledge modeling and reasoning. It remains a very complex field to explore and more semantic and syntactic features dealing with complexity of diagnostic criteria need to be further studied. Our efforts provide useful insight into how to develop a scalable, semantic-oriented and standards-based solution to support diagnostic criteria formalization and computerization.

## Additional files

Additional file 1: QDM elements used to build an AMI DCDO. An Excel spreadsheet file containing a list of QDM elements related to AMI diagnostic criteria, This is an example for illustrating the DCDO building. (XLSX 11 kb)
Additional file 2: HQMF templates used for coding AMI diagnostic criteria. A MS Word file containing 3 types of HQMF template used for coding AMI diagnostic criteria. These structured templates are used to support the automation for populating of an AMI DCDO. (DOCX 15 kb)
Additional file 3: An AMI DCDO in OWL. An OWL file representing a DCDO for AMI diagnostic criteria. (OWL 24 kb)

## Abbreviations

HQMF: Health quality measure format
ICD: International classification of diseases
QDM: Quality data model
SWRL: Semantic web rule language
WHO: World Health Organization

## Footnotes

1
https://vsac.nlm.nih.gov/
