# Supplementary material for: Developing a modular architecture for creation of rule-based clinical diagnostic criteria
Source: BioData Min. 2016 Oct 21;9:33. doi: 10.1186/s13040-016-0113-5 (PMC5073928; doi:10.1186/s13040-016-0113-5)
Supplement: Additional file 2: — HQMF templates used for coding AMI diagnostic criteria. A MS Word file containing 3 types of HQMF template used for coding AMI diagnostic criteria. These structured templates are used to support the automation for populating of an AMI DCDO. (DOCX 15 kb) [file 13040_2016_113_MOESM2_ESM.docx]

1. Laboratory Test, Result

hqmf r1 template - 2.16.840.1.113883.3.560.1.12

<act classCode="ACT" moodCode="EVN" isCriterionInd="true">

<templateId root="{$getTemplateOID}"/>

<id root="{$QDMElementUniqueId}"/>

<code code="30954-2" displayName="Results" codeSystem="2.16.840.1.113883.6.1">

<sourceOf typeCode="COMP">

<observation classCode="OBS" moodCode="EVN" isCriterionInd="true">

<code code="{$valueSetOID}" codeSystem="2.16.840.1.113883.3.560.101.1" displayName="{$displayName}"/>

<title>"{$datatypeName}: {$valueSetName}"</title>

<statusCode code="completed"/>

</observation>

</sourceOf>

</act>

1. Symptom, Active

hqmf r1 template - 2.16.840.1.113883.3.560.1.69

<act classCode="ACT" moodCode="EVN" isCriterionInd="true">

<templateId root="{$getTemplateOID}"/>

<id root="{$QDMElementUniqueId}"/>

<code code="46691-2" codeSystem="2.16.840.1.113883.6.1" displayName="Symptoms"/>

<sourceOf typeCode="COMP">

<observation classCode="OBS" moodCode="EVN" isCriterionInd="true">

<code code="{$valueSetOID}" codeSystem="2.16.840.1.113883.3.560.101.1" displayName="{$displayName}"/>

<title>"{$datatypeName}: {$valueSetName}"</title>

<sourceOf typeCode="REFR">

<observation classCode="OBS" moodCode="EVN" isCriterionInd="true">

<code code="33999-4" codeSystem="2.16.840.1.113883.6.1" displayName="Status"/>

<value xsi:type="CD" code="55561003" displayName="active" codeSystem="2.16.840.1.113883.6.96"/>

</observation>

</sourceOf>

</observation>

</sourceOf>

</act>

1. Diagnostic Study, Performed

hqmf r1 template - 2.16.840.1.113883.3.560.1.3

<act classCode="ACT" moodCode="EVN" isCriterionInd="true">

<templateId root="{$getTemplateOID}"/>

<id root="{$QDMElementUniqueId}"/>

<code code="30954-2" codeSystem="2.16.840.1.113883.6.1" displayName="Results"/>

<sourceOf typeCode="COMP">

<observation classCode="OBS" moodCode="EVN" isCriterionInd="true">

<code code="{$valueSetOID}" codeSystem="2.16.840.1.113883.3.560.101.1" displayName="{$displayName}"/>

<title>"{$datatypeName}: {$valueSetName}"</title>

<statusCode code="completed"/>

</observation>

</sourceOf>

</act>
